# The ISN/RPS 2016 classification predicts renal prognosis in patients with first-onset class III/IV lupus nephritis

**DOI:** 10.1038/s41598-020-78972-1

**Published:** 2021-01-15

**Authors:** Asaka Hachiya, Munetoshi Karasawa, Takahiro Imaizumi, Noritoshi Kato, Takayuki Katsuno, Takuji Ishimoto, Tomoki Kosugi, Naotake Tsuboi, Shoichi Maruyama

**Affiliations:** 1 Department of Nephrology, Nagoya University Graduate School of Medicine, Nagoya, Aichi Japan; 2 Center for Advanced Medicine and Clinical Research, Nagoya University Hospital, Nagoya, Aichi Japan; 3 Department of Nephrology and Rheumatology, Aichi Medical University, Nagakute, Aichi Japan; 4 Department of Nephrology, Fujita Health University Graduate School of Medicine, Toyoake, Aichi Japan

**Keywords:** Lupus nephritis, Rheumatic diseases

## Abstract

Lupus nephritis (LN) is a life-threatening complication of systemic lupus erythematosus. The 2003 pathological classification of LN was revised in 2016; it quantitatively evaluates the interstitium in addition to the glomeruli. We performed a retrospective multi-centre cohort study and investigated the utility of the 2016 classification—including the activity index (AI), chronicity index (CI), and each pathological component to predict complete remission or renal function decline, defined as 1.5-fold increase in serum creatinine levels—and compare with that of the 2003 classification. Ninety-one consecutive adult patients with first-onset class III/IV LN who were newly prescribed any immunosuppressants were enrolled and followed up for a median of 51 months from January 2004. Cox regression analysis demonstrated the subclasses based on the 2003 classification, which mainly evaluate glomerular lesions, were not associated with clinical outcomes. After adjustments for estimated glomerular filtration rate and urinary protein levels, higher CI and higher interstitial fibrosis and lower hyaline deposit scores were associated with renal functional decline. Similarly, higher CI and interstitial inflammation scores were associated with failure to achieve complete remission. Therefore, the 2016 classification can predict the clinical outcomes more precisely than the 2003 classification.

## Introduction

Systemic lupus erythematosus (SLE) is an autoimmune disease with a wide variety of clinical manifestations that can affect any organ. Approximately 50% of patients with SLE have lupus nephritis (LN) during the course of the disease, and up to 10% of patients with LN develop end-stage renal disease^1,2^. The mortality rate in patients with LN is higher than that in patients without LN^2,3^. Previous reports have demonstrated that renal function decline at baseline^4,5^ and delayed treatment responsiveness^6–8^ and were independent risk factors for poor renal prognosis. Therefore, it is crucial to identify factors that can predict early treatment responsiveness. According to the 2003 classification by the International Society of Nephrology/Renal Pathology Society (ISN/RPS), LN was classified into six classes based solely on the degree of glomerular injury based on renal histopathology^9^. Of the six classes, classes III and IV are especially important because of the high disease activity and poor renal prognosis in them^1^. The major scope of the 2003 classification was standardizing the definitions of pathologic findings, emphasizing clinically relevant lesions, and encouraging uniform and reproducible reporting across clinical centres. After the 2003 classification was published, various verification studies have demonstrated its clinical usefulness^10–13^ and the high interobserver reproducibility in diagnosing LN^14^. However, several studies have suggested that further improvements to the 2003 classification are needed^15–17^. Non-glomerular lesions, such as vascular^18^ and tubulointerstitial lesions^19–23^, which were not included in the 2003 classification were found to be important in predicting the prognosis in LN. Subsequently, the classification was revised by ISN/RPS in 2016 and published in 2018^24^.

One of the major changes in the 2016 classification was the introduction of the modified semi-quantitative scoring system that included activity index (AI) and chronicity index (CI), which were originally published in 1983^25^. AI and CI were introduced instead of subclass A, A/C, or C used for qualitative assessment of active or chronic lesions in the 2003 classification; subclass A was for purely active lesions, subclass A/C was for any combination of active and chronic lesion, subclass C was for purely chronic lesions^9^. AI includes pathological findings, such as endocapillary hypercellularity, neutrophils/karyorrhexis, fibrinoid necrosis, hyaline deposits, cellular and/or fibrocellular crescents, and interstitial inflammation. CI includes pathological findings, such as global/segmental sclerosis, fibrous crescents, interstitial fibrosis (IF), and tubular atrophy (TA). Of all these parameters, the scores of fibrinoid necrosis and cellular/fibrocellular crescents were set doubled weight. Notably, the 2016 classification incorporated the evaluation of tubulointerstitial lesions in the quantitative scoring system in the form of AI for interstitial inflammation and CI for IF/TA as opposed to the 2003 classification, which was merely based on the glomerular lesions. Several definitions of the pathological findings have also been revised. To date, the clinical utility of the 2016 classification has not been fully investigated.

The aim of the present study was to investigate the clinical usefulness of the 2016 classification with that of the 2003 classification by evaluating the achievement of complete remission (CR) and renal function decline in adult patients with first-onset class III/IV LN based on the Nagoya Kidney Disease Registry (N-KDR).

## Results

### Study participants

**Figure 1:**
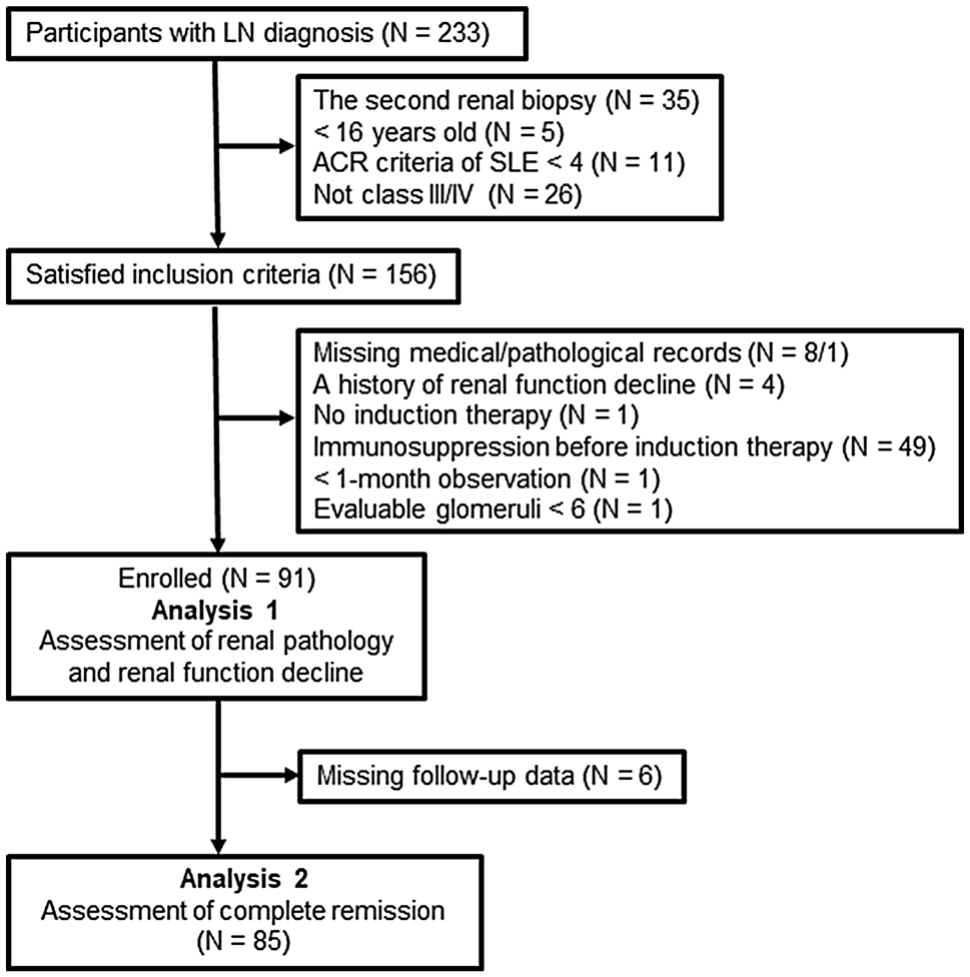
Flow chart of patient selection. Ninety-one patients with first-onset lupus nephritis were enrolled in this study and assessed for pathological findings and renal function decline during the observational duration (Analysis 1). After excluding 6 patients with missing follow-up data, achievement of complete remission was evaluated for 85 patients (Analysis 2). *LN* lupus nephritis; *ACR* American College of Rheumatology.

We screened 233 consecutive patients with LN in our real biopsy registry between January 2004 and December 2014. We enrolled patients who underwent the first renal biopsy, were ≥ 16 years of age, who met ≥ 4 American College of Rheumatology (ACR) criteria^26^ of SLE, and were classified to have class III or IV LN. We excluded patients with missing medical or pathological records (n = 9), a history of renal function deterioration (n = 4), conservative treatment without immunosuppressive therapy (n = 1), immunosuppression before induction therapy for LN (n = 49), observational period less than a month (n = 1), and evaluable glomeruli less than six (n = 1). Finally, 91 patients were enrolled in this study. We assessed their pathological findings and renal function decline during the observational duration (Analysis 1). Of these, six patients were excluded because of missing adequate follow-up data, and 85 were assessed for CR (Analysis 2). The detailed flowchart is shown in Fig. 1.

### Baseline characteristics

Baseline characteristics are summarized according to the eGFR^27^ levels at baseline as lower eGFR group (eGFR < 60 ml/min/1.73 m^2^, n = 42 [46%]) and higher eGFR group (eGFR ≥ 60 ml/min/1.73 m^2^, n = 49 [54%]) (Table 1). Patients in the lower eGFR group were older, had heavier proteinuria, more severe haematuria and higher proportion of nephrotic syndrome than those in the higher eGFR group. Anti-dsDNA, serum C3 levels, and SLE disease activity index (SLEDAI)^28^ scores were not significantly different between the groups.

**Table 1:** Baseline characteristics (N = 91).

	All (N = 91)	eGFR < 60 (N = 42)	eGFR ≥ 60 (N = 49)	*p* value
Female, N (%)	65 (71)	33 (79)	32 (65)	0.16
Age at diagnosis, years old, Median [IQR]	47 [30–62]	57 [34–69]	41 [25–59]	0.007
Serum creatinine, mg/dl, Median [IQR]	0.89 [0.65–1.16]	1.18 [1.00–1.50]	0.66 [0.57–0.85]	< 0.001
eGFR, ml/min/1.73 m^2^, Median [IQR]	64 [45–84]	43 [29–51]	80 [70–106]	< 0.001
Anti-dsDNA antibody level, IU/ml, Median [IQR]	96 [19–292]*	37 [17–144]**	132 [38–342]***	0.08
Serum C3 level, mg/dl, Median [IQR]	40 [28–60]	42 [28–64]	38 [28–57]	0.50
Urinary protein, g/day or g/gCr, Median [IQR]	1.9 [0.9–4.6]****	3.3 [1.5–5.3]	1.3 [0.7–2.9]****	0.001
Haematuria, N (%)				
−	19 (20)	2 (5)	17 (35)	0.002
+	12 (13)	4 (10)	8 (16)	
++	18 (20)	11 (26)	7 (14)	
+++	42 (46)	25 (60)	17 (35)	
Nephrotic syndrome, N (%)	38 (42)	24 (57)	14 (29)	0.006
SLEDAI score, Median [IQR]	19 [16–22]	19 [16–21]	18 [16–24]	0.92

Number of missing data: *N = 27, **N = 10, ***N = 17, ****N = 1.

*N* number; interquartile range, IQR, *eGFR* estimated glomerular filtration rate, *SLEDAI* systemic lupus erythematosus disease activity score.

### Pathological findings according to the 2003/2016 classification

**Figure 2:**
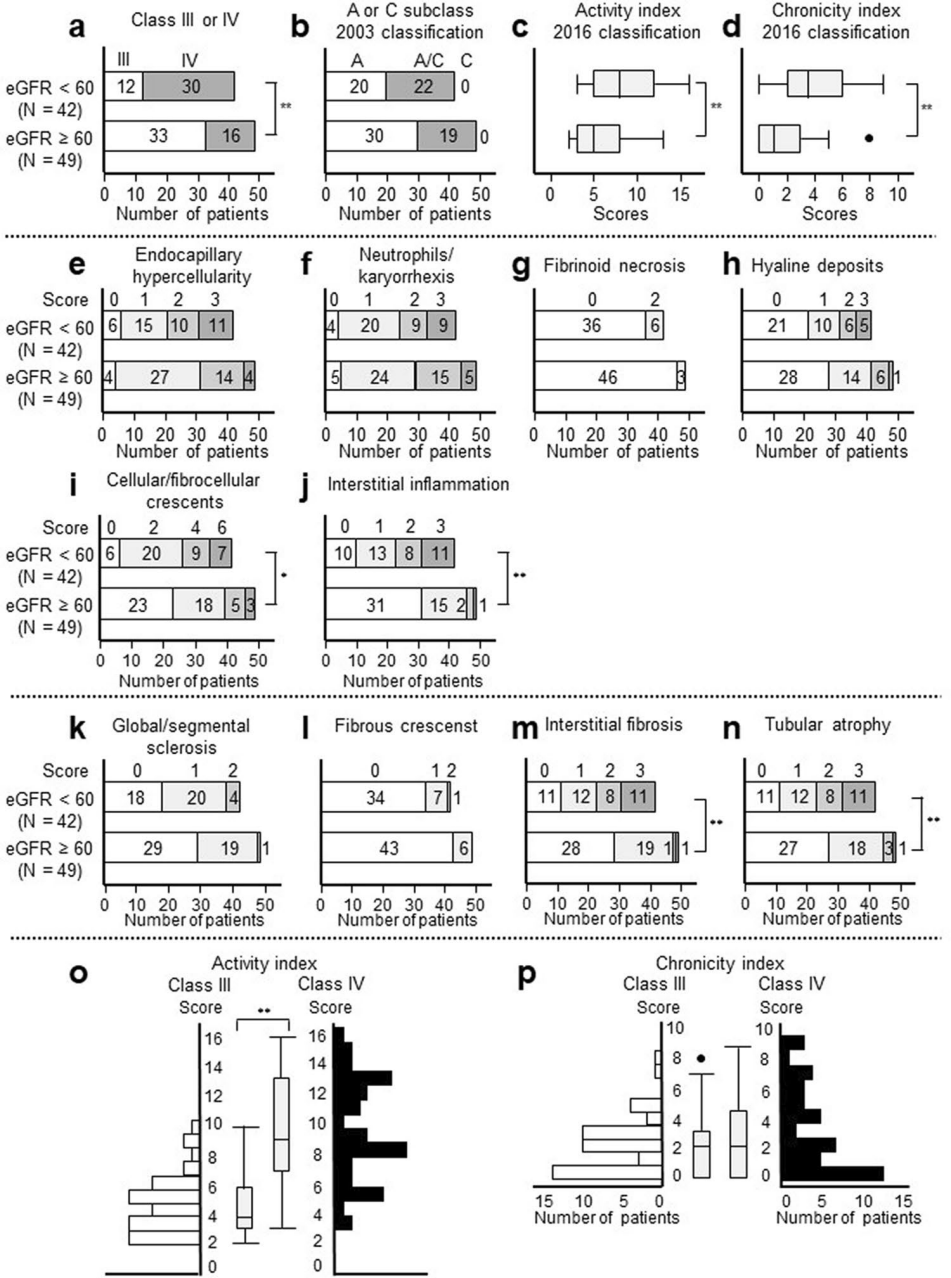
Pathological findings according to the 2003/2016 classification. Baseline pathological findings are described according to the baseline eGFR levels (eGFR < 60 ml/min/1.73 m^2^, n = 42 and eGFR ≥ 60 ml/min/1.73 m^2^, n = 49). (**a**,**b**) are based on the 2003 classification: class III or IV (**a**) and subclass of A, C, and A/C (**b**). (**c**,**d**) are based on the 2016 classification: activity index (**c**) and chronicity index (**d**). (**e**–**j**) represent the following pathological components of activity index in the 2016 classification: endocapillary hypercellularity (**e**), neutrophils/karyorrhexis (**f**), fibrinoid necrosis (**g**), hyaline deposits (**h**), cellular/fibrocellular crescents (**i**), and interstitial inflammation (**j**). (**k**–**n**) represent chronicity index in the 2016 classification: global/segmental sclerosis (**k**), fibrous crescents (**l**), interstitial fibrosis (**m**), and tubular atrophy (**n**). (**o**,**p**) are distributions of activity index (**o**) and chronicity index (**p**) in patients with class III and IV LN, respectively. *N* number, *eGFR* estimated glomerular filtration rate, *LN* lupus nephritis. *p < 0.01. **p < 0.001.

The proportion of the patients with class IV LN was higher in the lower eGFR group than that in the higher eGFR group (71% [30/42] and 33% [16/49], respectively) (Fig. 2a), while there was no difference in the A and A/C subclasses (Fig. 2b). Both AI and CI were higher in the lower eGFR group (Fig. 2c,d) than those in the higher eGFR group. In the pathological components of AI (Fig. 2e–j), patients in the lower eGFR group had higher scores of cellular/fibrocellular crescents (Fig. 2i) and interstitial inflammation (Fig. 2j) than those in the higher eGFR group. In the pathological components of CI (Fig. 2k–n), patients in the lower eGFR group had higher scores of IF (Fig. 2m) and TA (Fig. 2n) than those in the higher eGFR group. Of all the pathological components, fibrinoid necrosis of 4 or 6 points and global/segmental sclerosis and fibrous crescents with 3 points was not observed in any of the patients (Fig. 2g,k,l). We analysed the relationship between AI/CI and class III/IV LN. The median AI in class IV was higher than that in class III (9 [interquartile range, IQR: 7–13] vs. 4 [IQR: 3–6], respectively). All patients with ≥ 11 points in AI had pathological class IV (n = 19) (Fig. 2o). The median CI in both class III and IV was 2 points, and there was no statistically significant difference between the classes (Fig. 2p).

### Correlation between the baseline characteristics and pathological findings

AI was inversely correlated with eGFR (Spearman’s correlation: Rs = − 0.40) and directly with urinary protein levels (Rs = 0.36), severity of haematuria (Rs = 0.40) and anti-dsDNA antibody level (Rs = 0.35). CI was inversely correlated with eGFR (Rs = − 0.52). Endocapillary hypercellularity was inversely correlated with serum C3 levels (Rs = − 0.43). Cellular crescents were inversely correlated with eGFR (Rs = − 0.34). Interstitial inflammation and IF/TA were also inversely correlated with eGFR (Rs = − 0.55, − 0.56, and − 0.53, respectively) (Table 2). AI was highly correlated with the scores of cellular/fibrocellular crescents (Rs = 0.84). CI was strongly correlated with the scores of interstitial inflammation (Rs = 0.91), IF (Rs = 0.95), and TA (Rs = 0.95). They demonstrated high correlation with each other as well (interstitial inflammation and IF, Rs = 0.93; interstitial inflammation and TA, Rs = 0.95; and IF and TA, Rs = 0.98) (see Supplementary Table S1 online).

**Table 2:** Correlations between the baseline characteristics and pathological findings.

	eGFR, ml/min/1.73 m^2^	Urinary protein^†^, g/day or g/gCr	Haematuria*	Anti-dsDNA antibody level^††^, IU/ml	Serum C3 level, mg/dl
**Activity index**	− 0.40	0.36	0.40	0.35	− 0.21
Endocapillary hypercellularity	− 0.09	0.37	0.33	0.30	− 0.43
Neutrophils/karyorrhexis	− 0.06	0.21	0.33	0.28	− 0.20
Hyaline deposits	− 0.17	0.19	0.26	0.32	− 0.31
Fibrinoid necrosis	− 0.03	0.07	0.06	0.16	− 0.04
Cellular/fibrocellular crescents	− 0.34	0.24	0.27	0.19	− 0.03
Interstitial Inflammation	− 0.55	0.15	0.14	0.05	0.13
**Chronicity index**	− 0.52	0.11	0.07	0.05	0.19
Global/segmental sclerosis	− 0.26	− 0.02	0.00	− 0.05	0.17
Fibrous crescents	− 0.06	− 0.06	− 0.06	0.07	0.07
Interstitial fibrosis	− 0.56	0.17	0.10	0.05	0.18
Tubular atrophy	− 0.53	0.13	0.08	0.06	0.16

Correlations between the baseline clinical data and pathological scores are described by using Spearman correlation coefficients.

*eGFR* estimated glomerular filtration rate.

Number of missing data: ^†^N = 1, ^††^N = 27.

*Divided into four categorical variables depending on the severity; − (0), + (1), ++ (2), +++ (3).

### Medications during the induction therapy, clinical outcomes, and adverse events

The overall median observation period was 51 (IQR: 23–77) months, and there was no statistically significant difference between the groups (*p* = 0.50) (Table 3). Median interval from renal biopsy to start of the induction therapy was 1 (IQR: − 7–9) day, and there was no statistically significant difference between the two groups (*p* = 0.85). During induction therapy, prednisolone was prescribed for all patients. The proportion of patients in the lower eGFR group who received methylprednisolone pulse therapy was higher than that in the higher eGFR group (62% [26/42] vs. 51% [25/49], respectively). However, the proportion of patients who received any type of immunosuppressants was not statistically different between the two groups. Of all patients, five were lost to follow-up and four died during induction therapy. Of the remaining, 82 received maintenance therapy, and of these, 66 responded to induction therapy^29^. There was no statistically significant difference in the content of maintenance treatment between the groups. Overall, 54/85 patients achieved CR; the cumulative incidence of CR in the lower eGFR group was lower (38%, 15/39) than that in the higher eGFR group (55%, 39/46). Overall (n = 91), 16 patients developed 1.5-fold increase in sCr, eight patients had doubling of sCr, and two patients reached end-stage renal disease (ESRD) during the entire observation period. Six patients died, and all of them were in the lower eGFR group. Regarding the adverse events after the initiation of induction therapy, the incidence of steroids-induced diabetes was significantly higher in the lower eGFR group (52%, 22/42) than that in the higher eGFR group (31%, 15/46) (see Supplementary Table S2 online).

**Table 3:** Medication during 6-month induction therapy and clinical outcomes.

	All (N = 91)	eGFR < 60 (N = 42)	eGFR ≥ 60 (N = 49)	*p* value
**Follow-up duration, month, Median [IQR]**	51 [23–77]	53 [13– 80]	51 [31–76]	0.50
**Days from renal biopsy to initial medication, Median [IQR]**	1 [− 7–9]	1 [− 7–8]	0 [− 7–8]	0.85
**Medication during 6-month induction therapy**				
Prednisolone, N (%)	91 (100)	42 (100)	49 (100)	
Methyl prednisolone pulse therapy, N (%)	51 (56)	26 (62)	25 (51)	0.002
Calcineurin inhibitor, N (%)	43/91 (47)	19/42 (45)	24/49 (49)	0.72
Cyclophosphamide, N (%)	21/91 (23)	11/42 (26)	10/49 (20)	0.51
*Azathioprine, N (%)	11/91 (12)	7/42 (17)	4/49 (8)	0.22
Mizoribine, N (%)	22/91 (24)	7/42 (17)	15/49 (31)	0.12
*Mycophenolate mofetil, N (%)	8/91 (9)	2/42 (5)	6/49 (12)	0.21
Rituximab, N (%)	1/91 (1)	0/42 (0)	1/49 (2)	0.35
**Medication after 6-month induction therapy (maintenance therapy)**				
Prednisolone, N (%)	82/82 (100)	36/36 (100)	46/46 (100)	
Calcineurin inhibitor, N (%)	32/82 (39)	13/36 (36)	19/46 (41)	0.63
Cyclophosphamide, N (%)	1/82 (1)	0/36 (0)	1/46 (2)	0.37
Azathioprine, N (%)	10/82 (12)	5/36 (14)	5/46 (11)	0.68
Mizoribine, N (%)	18/82 (22)	6/36 (17)	12/46 (26)	0.31
Mycophenolate mofetil, N (%)	8/82 (10)	1/36 (3)	7/46 (15)	0.06
**Clinical outcomes**				
Complete remission, N (%)	54/85 (65)	15/39 (38)	39/46 (55)	< 0.001
1.5-fold increase in serum creatinine, N (%)	16 (18)	8 (19)	8 (16)	0.73
Doubling of serum creatinine, N (%)	8 (9)	5 (12)	3 (6)	0.33
End-stage renal disease, N (%)	2 (2)	1 (3)	1 (2)	0.91
Death, N (%)	6 (7)	6 (15)*	0 (0)	0.006

*N* number, *eGFR* estimated glomerular filtration rate, *IQR* interquartile range.

*Cause of death; Sepsis (N = 3), Pneumonitis (N = 1), Upper gastrointestinal bleeding (N = 1), Cerebral breeding (N = 1).

### Survival curves for renal function decline and CR

**Figure 3:**
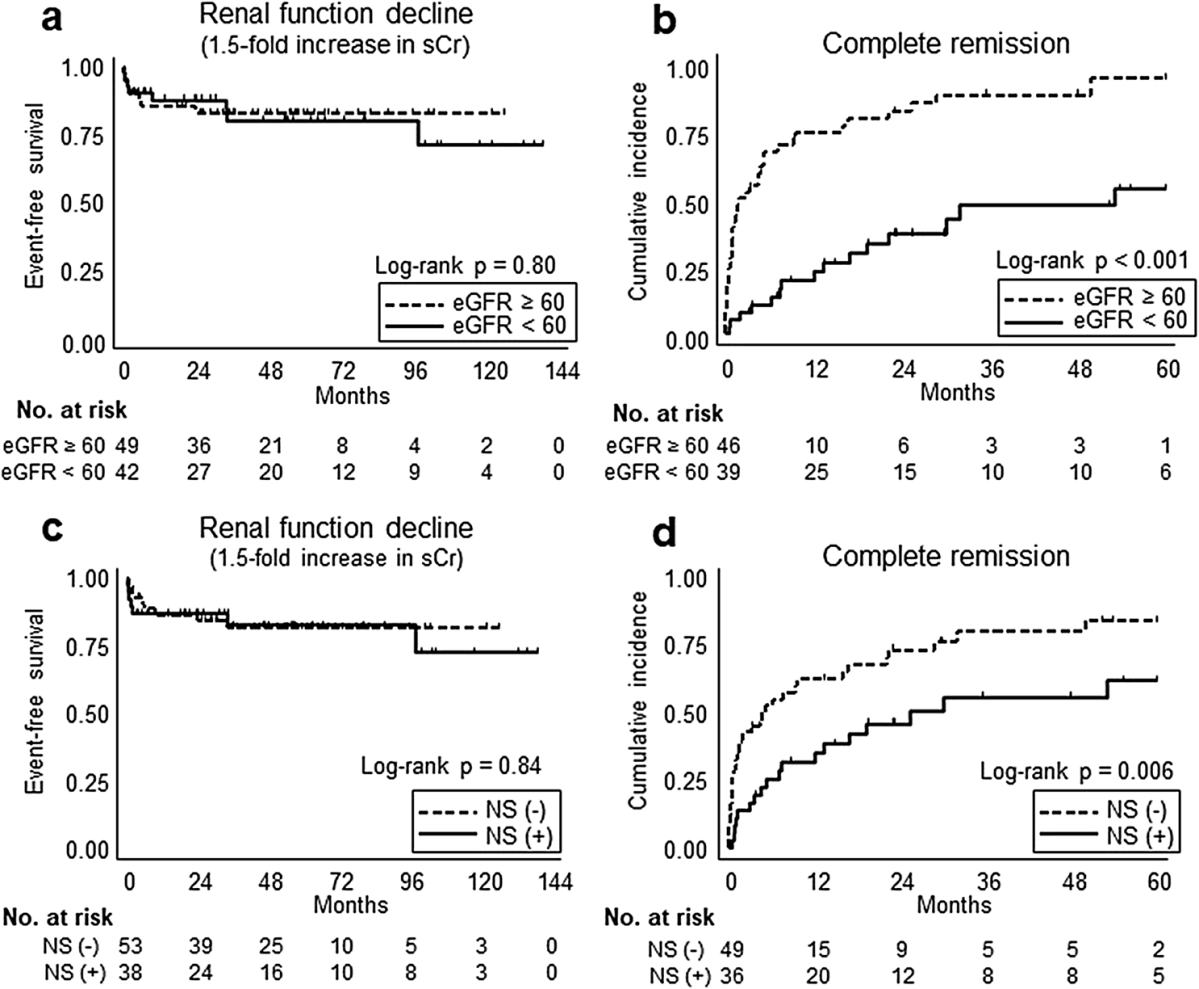
Survival curves of clinical outcomes. Kaplan–Meier plots are described according to the baseline eGFR levels (**a**,**b**), and with or without nephrotic syndrome (**c**,**d**). The cumulative incidence of renal event (1.5-fold increase in serum creatinine, sCr)-free survival is indicated on the y-axis (**a**,**c**), and that of complete remission is indicated on the y-axis (**b**,**d**). Duration (months) from the initiation of the induction therapy is indicated on x-axis. *eGFR* estimated glomerular filtration rate; *NS* nephrotic syndrome.

The cumulative incidence of renal event (1.5-fold increase in sCr)-free survival and CR are illustrated in Fig. 3. Time to CR was assessed within 5 years from the initiation of induction therapy because none of the patients achieved CR after 5 years. The baseline eGFR levels were not associated with renal function decline (*p* = 0.80) (Fig. 3a), but patients in the higher eGFR group were more likely to achieve CR than were those in the lower eGFR group (*p* < 0.001) (Fig. 3b). Similarly, the presence of nephrotic syndrome was not associated with renal function decline (*p* = 0.84) (Fig. 3c), but patients without nephrotic syndrome were also more likely to achieve CR than those with nephrotic syndrome (*p* = 0.006) (Fig. 3d).

### Clinical predictors of renal function decline

Baseline disease activity metrics (i.e. eGFR less than 60 ml/min/1.73 m^2^, existence of nephrotic syndrome, and levels of anti-dsDNA antibody and serum C3 levels) were not associated with renal function decline (Table 4). Patients with class IV were not significantly different in terms of renal function decline from those of class III (hazards ratio, HR [95% confident interval, CI] 0.58 [0.21–1.60]). Similarly, patients of A/C subclass were also not statistically different from those of A subclass (HR [95% CI] 0.90 [0.33–2.42]). Regarding the 2016 classification, higher CI was associated with renal function decline (HR [95% CI] 1.18 [0.99–1.40]), although higher AI was not associated with it (HR [95% CI] 1.00 [0.88–1.15]. Higher CI was identified as an independent predictor of renal function decline after adjusting for eGFR and urinary protein level (adjusted HR [95% CI] 1.24 [1.01–1.53]) (Model 1 in Table 4). Higher scores of IF and lower scores of hyaline deposits, which were chosen via the forward–backward stepwise selection method, were identified as independent predictors of renal function decline (adjusted HR [95% CI] 2.66 [1.43–4.93], 0.45 [0.21–0.97], respectively). Scores of global/segmental sclerosis were not associated with renal function decline after adjustments for eGFR, urinary protein levels, and pathologically relevant factors (adjusted HR [95% CI] 0.40 (0.14–1.13) (Model 2 in Table 4).

**Table 4:** Associated factors for renal function decline.

Factors	Univariable analysis		Multivariable analysis			
			Model 1*		Model 2**	
	HR [95% CI]	*p* value	Adjusted HR [95% CI]	*p* value	Adjusted HR [95% CI]	*p* value
**Baseline disease activity**						
eGFR < 60 ml/min/1.73m^2^, vs. ≥ 60	1.13 [0.42–3.04]	0.80	0.68 [0.19–2.41]	0.55	0.44 [0.10–1.88]	0.27
Nephrotic syndrome, vs. without	1.11 [0.48–1.35]	0.28	1.36 [0.43–4.30]	0.60	0.97 [0.28–3.40]	0.96
Anti-dsDNA antibody level, IU/ml ^† ‡^	1.14 [0.48–2.66]	0.77				
Serum C3 level, mg/dl	1.01 [0.99–1.03]	0.54				
**Pathological class**						
Class IV, vs. Class III	0.58 [0.21–1.60]	0.29				
**The 2003 classification**						
Subclass A/C, vs. A	0.90 [0.33–2.42]	0.88				
**The 2016 classification**						
Activity index, per 1 point	1.00 [0.88–1.15]	0.72	0.96 [0.81–1.13]	0.62		
Chronicity index, per 1 point	1.18 [0.99–1.40]	0.047	1.24 [1.01–1.53]	0.043		
**Pathological findings**						
*Active lesion*						
Endocapillary hypercellularity, per 1 point	0.53 [0.28–1.00]	0.06				
Neutrophils/karyorrhexis, per 1 point	1.12 [0.63–1.96]	0.70				
Hyaline deposits, per 1 point	0.47 [0.21–1.05]	0.06			0.45 [0.21–0.97]	0.042
Fibrinoid necrosis, per 2 points	1.25 [0.28–5.58]	0.91				
Cellular/fibrocellular crescents, per 2 points	1.13 [0.68–1.88]	0.35				
Interstitial inflammation, per 1 point	1.67 [1.10–2.52]	0.010				
*Chronic lesion*						
Global/segmental sclerosis, per 1 point	0.75 [0.31–1.80]	0.81			0.40 [0.14–1.13]	0.08
Fibrous crescents, per 1point	0.77 [0.19–3.10]	0.95				
Interstitial fibrosis, per 1 point	1.73 [1.14–2.64]	0.012			2.66 [1.43–4.93]	0.002
Tubular atrophy, per 1 point	1.70 [1.11–2.61]	0.018				

*eGFR* estimated glomerular filtration rate, *HR* hazard ratio, *CI* confidence interval.

*Model 1 is adjusted by activity index and chronicity index by the baseline eGFR levels (cut-off of 60 ml/min/1.73 m^2^) and the presence of nephrotic syndrome.

**Model 2 is adjusted by pathological components by eGFR and urinary protein levels. Pathological variables associated with 1.5-fold increase in serum creatinine were identified on forward–backward stepwise regression analysis.

^†^Number of missing data: N = 27.

^‡^Log-transformed.

### Identification of clinical predictors of CR

Baseline renal function decline was associated with achieving CR, while nephrotic syndrome, anti-dsDNA antibody, and serum C3 levels were not (Table 5). Patients with class IV LN were not significantly different in terms of achieving CR than those with class III LN (HR [95% CI] 0.67 [0.39–1.15]). Similarly, patients with A/C subclass were not significantly different from those with A subclass (HR [95%CI] 0.82 [0.48–1.40]). Regarding the 2016 classification, higher AI or CI was associated with failure in achieving CR (HR [95%CI] 0.89 [0.82–0.96] vs. 0.70 [0.67–0.82], respectively). AI/CI was adjusted for clinically relevant factors, such as baseline eGFR levels and presence of nephrotic syndrome (Model 1 in Table 5). The association between AI and CR was no longer significant after adjustments for eGFR and urinary protein levels (adjusted HR [95%CI] 0.99 [0.91–1.08]). Higher CI was identified as an independent predictor of failure in achieving CR (adjusted HR [95%CI] 0.75 [0.64–0.88]). Cellular crescents were associated with CR; however, they were not selected by the forward–backward stepwise selection method. The scores of interstitial inflammation were also adjusted for eGFR and urinary protein levels (Model 2 in Table 5. Higher interstitial inflammation score was identified as an independent predictor of failure in achieving CR (adjusted HR [95%CI] 0.39 [0.25–0.61]).

**Table 5:** Associated factors for complete remission.

Factors	Univariable analysis		Multivariable analysis			
			Model 1*		Model 2**	
	HR [95% CI]	*p* value	Adjusted HR [95% CI]	*p* value	Adjusted HR [95% CI]	*p* value
**Baseline disease activity**						
eGFR < 60 ml/min/1.73 m^2^, versus ≥ 60	0.22 [0.12–0.41]	< 0.001	0.39 [0.20–0.76]	0.006	0.41 [0.21–0.80]	0.009
Nephrotic syndrome, vs. without	0.46 [0.26–0.81]	0.007	0.69 [0.36–1.32]	0.26	0.79 [0.43–1.44]	0.44
Anti-dsDNA antibody level, IU/ml^†‡^	1.00 [0.64–1.57]	1.00				
Serum C3 level, mg/dl	1.00 [0.99–1.01]	0.85				
**Pathological class**						
Class IV, versus Class III	0.67 [0.39–1.15]	0.15				
**The 2003 classification**						
Subclass A/C, versus A	0.82 [0.48–1.40]	0.46				
**The 2016 classification**						
Activity index, per 1 point	0.89 [0.82–0.96]	0.003	0.99 [0.91–1.08]	0.90		
Chronicity index, per 1 point	0.70 [0.67–0.82]	< 0.001	0.75 [0.64–0.88]	< 0.001		
**Pathological findings**						
*Active lesion*						
Endocapillary hypercellularity, per 1 point	1.03 [0.77–1.37]	0.86				
Neutrophils/karyorrhexis, per 1 point	0.88 [0.65–1.19]	0.39				
Hyaline deposits, per 1 point	1.01 [0.78–1.30]	0.95				
Fibrinoid necrosis, per 2 points	0.54 [0.19–1.49]	0.23				
Cellular/fibrocellular crescents, per 2 points	0.70 [0.51–0.95]	0.024				
Interstitial inflammation, per 1 point	0.34 [0.22–0.52]	< 0.001			0.39 [0.25–0.61]	< 0.001
*Chronic lesion*						
Global/segmental sclerosis, per 1 point	0.68 [0.43–1.09]	0.11				
Fibrous crescents, per 1point	0.66 [0.31–1.41]	0.28				
Interstitial fibrosis, per 1 point	0.38 [0.26–0.57]	< 0.001				
Tubular atrophy, per 1 point	0.38 [0.26– 0.56]	< 0.001				

*eGFR* estimated glomerular filtration rate, *HR* hazard ratio, *CI* confidence interval.

*Model 1 is adjusted by activity index and chronicity index by the baseline eGFR levels (cut-off of 60 ml/min/1.73 m^2^) and the presence of nephrotic syndrome.

**Model 2 is adjusted by a pathological component by eGFR and urinary protein levels. Pathological variables associated with complete remission were identified on forward–backward stepwise regression analysis.

^†^Number of missing data: N = 29.

^‡^log-transformed.

## Discussion

We demonstrated the clinical usefulness of the 2016 classification based on a multivariable model approach, in which clinically relevant factors, such as eGFR and urinary protein levels were taken into consideration. Detailed analysis of the 2016 classification allowed us to better comprehend the clinical importance of evaluating the interstitial lesions. This is the first study to evaluate the utility of the 2016 classification in patients with first-onset class III/IV LN by comparisons with the 2003 classification in terms of predicting clinically important outcomes, CR, and renal function decline.

In the present study, CI was associated with renal function decline and CR independently of eGFR and urinary protein levels mainly due to its high correlation with the scores of interstitial lesions. Both AI and CI were predictive of CR. Of the components of AI, interstitial inflammation was associated with CR, and of the components of CI, IF was independently associated with renal function decline. Therefore, it is crucial to assess interstitial lesions in order to predict renal prognosis in patients with LN. In contrast, AI was not associated with CR after adjusting for eGFR and urinary protein levels. Cellular crescents, which were highly correlated with AI, had moderate correlation with eGFR and urine protein levels. These correlations probably attenuate the association of AI and CR. Therefore, we demonstrated the utility of CI and importance of assessing interstitial regions in predicting renal prognosis, as previously reported^19–23^.

In our study, however, we did not identify active glomerular lesions as potential risk factors of poor renal prognosis. Crescentic lesions and fibrinoid necrosis were not associated with renal function decline in our study, although previous reports showed them as indicators for poor renal prognosis^4,19,23,29,30^. Hyaline deposits were rather inversely correlated with renal function decline in the present study while Austin et al.^25^ adopted it as an active indicator associated with prognosis. A recent research for clinical and histopathologic predictors of renal outcomes for LN demonstrated that wire loops, or hyaline deposits, were associated with eGFR recovery rather than decline^23^. This is consistent with our results. There are two possible reasons for these discrepancies. One is the improvement of treatment for LN over time. Our patients received immunosuppressant therapy depending on their disease activities, and as high as 80.5% of them responded to the treatments accordingly. Of active glomerular lesions, hyaline deposits, or subendothelial deposits, might represent an early pathological change of LN that was likely to heal easily by immunosuppressive treatment. Another reason is the differences in the background of patients. Most of the previous studies included first-onset LN patients as well as those who had already been treated for SLE. In contrast, we included only first-onset LN patients without previous immunosuppressive treatments. Because active glomerular lesions of LN were considered to be reversible, we believe that they did not reflect the long-term renal prognosis in our study.

We suggest that the pathological classification system should be improved by investigating the effects of each pathological component through an evidence-based process such as the MEST score in the Oxford classification of IgA nephropathy^31^. Our results suggest that treatment resistance factors, such as interstitial lesions and treatment response factors, such as hyaline deposits should be considered separately. Further investigations are required to identify the pathological findings that are associated with the clinical outcomes and determine their weightages in the scoring system.

There were several limitations to this study. First, this was a retrospective observational study. However, to the best of our knowledge, this is the largest multi-centre cohort study of adult patients with first-onset class III/IV LN. These results can be generalizable in various clinical settings. Second, there might have been substantial differences in the treatment strategies between the hospitals. There was no unified protocol for the treatment, and it was decided at the discretion of the doctors. The potential differences in the treatment strategies over the course of the study period might have also affected the clinical course of LN. However, these results reflect the real-world data and have high generalizability.

In conclusion, we demonstrated that comprehensive and quantitative assessments of the renal biopsy specimen based on the 2016 classification can provide useful information to predict the renal prognosis in patients with first-onset class III/IV LN. Of the pathological findings, interstitial lesions were strong predictors of both short- and long-term renal prognoses. Further prospective validation studies are currently underway.

## Methods

### Patient selection and study design

This study was a retrospective, multi-center cohort study. Primary LN was diagnosed in 233 consecutive patients from N-KDR between January 2004 and December 2014. Inclusion criteria were as follows: (1) diagnosed at first-biopsy, (2) aged over 16 years, (3) fulfilled 4 and more ACR criteria^26^, and (4) diagnosed with class III/IV LN. Exclusion criteria were as follows: (1) no medical or pathological records, (2) history of renal function decline, (3) no induction therapy, (4) previous immunosuppression, (5) less than 1-month observation period, and (6) total evaluable number of glomeruli less than 6. A history of renal function deterioration was defined as follows: (1) renal atrophy at diagnosis or (2) continuous decline in estimated glomerular filtration rate (eGFR) < 60 ml/min/1.73 m^2^ within 3 months prior to diagnosis. Induction therapy was defined as the 6-month immunosuppressive medications for remission induction for LN. Previous immunosuppression was defined as history of other immunosuppressive therapies before ≥ 2 weeks of initiation of induction therapy for LN. Overall, 91 patients with first-onset class III/IV LN and new prescriptions of any immunosuppression were observed between January 2004 and July 2016; the observations were performed until ESRD or death, whichever was early, or the last available data of urinary proteins or sCr. All of them were followed up at the following 16 nephrology centres: Nagoya University Hospital, Anjyo Kosei Hospital, Ogaki Municipal Hospital, Kasugai Municipal Hospital, Ichinomiya Municipal Hospital, Konan Kosei Hospital, Japanese Red Cross Nagoya Daiichi Hospital, Yokkaichi Municipal Hospital, Handa City Hospital, Tosei General Hospital, Chubu Rosai Hospital, Chutoen General Medical Center, Toyota Kosei Hospital, Gifu Prefectural Tajimi Hospital, Tsushima City Hospital, and Nagoya Memorial Hospital. All patients provided written informed consent. The study was approved by the Ethics Committee of the Nagoya University (approval number: 2010-1135-4) and adhered to the Declaration of Helsinki.

### Baseline characteristics

The baseline was defined as the time just prior to the initiation of induction therapy for LN. The clinical data included the sex, age, sCr, eGFR, which was estimated using the equation recently proposed by the Japanese Society of Nephrology: eGFR [ml/min/1.73 m^2^]0.194 × sCr^−1.094^ × Age^−0.287^ × 0.739 [if female]^27^, anti-dsDNA antibody level, serum C3 level, 24-h urinary protein excretion (g/day) or urinary protein-to-creatinine ratio (g/gCr), haematuria, and SLEDAI score^28^. The severity of haematuria expressed as −/+/++/+++. Nephrotic syndrome was defined as urinary protein ≥ 3.5 g/day or urinary protein-to-Cr ratio ≥ 3.5, and serum albumin < 3.0 mg/dl.

### Pathological findings

All the patients (n = 91) were assessed renal pathological findings which were assessed according to the ISN/RPS 2003^9^ and 2016^24^ classifications. All of the biopsy samples from 16 facilities were processed at the department of Nephrology in Nagoya University Hospital. Renal biopsy specimens were evaluated under light microscopy separately by two nephrologists (A.H and M.K) under the supervision of one experienced nephropathologist (M.N). The stains used included periodic acid Schiff (PAS), periodic acid-methenamine-silver (PAMS), and Masson’s trichrome stains. In cases of conflicting interpretations, conclusion was derived based on discussions. The scores of AI and CI were calculated based on the 2016 classification.

### Medications during induction and maintenance therapy

All drugs used during induction and maintenance therapy were investigated. Induction therapy was defined as the immunosuppressive therapy for the first 6-month of treatment for LN. Maintenance therapy was defined as the immunosuppressive therapies administered after the 6-month induction therapy. The drugs included prednisolone, methyl prednisolone pulse, calcineurin inhibitors (cyclosporine or tacrolimus), cyclophosphamide, azathioprine, mizoribine, mycophenolate mofetil and rituximab.

### Adverse events

Adverse events after the initiation of induction therapy included cardiovascular disease, cerebrovascular disease, femoral head osteonecrosis, steroids-induced diabetes, gastric ulcers, first infectious disease that required hospitalization, herpes zoster or cytomegalovirus infections that required medications, and cancer. Steroids-induced diabetes was defined as initiating new antidiabetic medications after the initiation of induction therapy.

### Definition of clinical outcomes

The primary outcome was renal function decline, which was defined as 1.5-fold increase in sCr or 50% increase in sCr from the baseline level. The secondary outcome was the achievement of CR, which was defined as achievement of both proteinuria < 0.5 g/gCr or g/24 h and recovery of normal renal function^32^. Normal renal function was defined as (1) returning to the sCr levels before the onset of LN or (2) sCr < 1.0 mg/dl (if male) and < 0.7 mg/dl (if female) if the past sCr level was unknown. Treatment response to induction therapy was assessed at 6 months after the initiation of induction therapy, which was defined as both ≥ 50% decrease in proteinuria from the baseline to at least sub-nephrotic levels and stabilization (± 25%) or improvements in sCr (but not completely reverting to normal)^33^. Doubling of sCr was defined doubling of sCr level from the baseline value. ESRD was defined as the disease stage that required initiation of dialysis or renal transplantation.

### Statistical analysis

Continuous variables with asymmetric distribution are presented as median [IQR]. Categorical variables are expressed as percentages. Spearman’s correlation coefficients were used to examine the relationships between the continuous variables. The cumulative probability of attaining the outcomes was calculated using the Kaplan–Meier method, and log-rank test was employed for hypothesis testing. The time-to-clinical outcomes were calculated between the date of the initiation of induction therapy and the date of the clinical outcomes. Loss to follow-up, ESRD, and all-cause death were censored. In order to use the 2016 classification for quantitative prognostic evaluation, we performed exploratory investigation of their mutual correlation and relevance to the renal prognosis using Rs. The proportional hazards assumption for covariates was tested using scaled Schoenfeld residuals. Both baseline and pathological data were examined using univariable and multivariable Cox’s proportional hazards models in order to identify independent predictors associated with the clinical outcomes. Covariates included both the clinical and pathological findings, and we selected pathological components using a stepwise method to avoid multicollinearity of these findings. All statistical models were performed using complete case analysis. The level of statistical significance was set at *p* value < 0.05. All statistical analyses were performed using Stata SE v14.0 (STATA Corp, 4905 Lakeway Drive College Station, Texas 77845-4512, USA, www.stata.com).

## Supplementary information

Supplementary Tables.

## Data Availability

The data that support the findings of this study are available from the corresponding author upon reasonable request.
