# Supplementary material for: The ISN/RPS 2016 classification predicts renal prognosis in patients with first-onset class III/IV lupus nephritis
Source: Sci Rep. 2021 Jan 15;11:1525. doi: 10.1038/s41598-020-78972-1 (PMC7810677; doi:10.1038/s41598-020-78972-1)
Supplement: Supplementary file 1 — Supplementary Tables. [file 41598_2020_78972_MOESM1_ESM.pdf]

The ISN/RPS 2016 classification predicts renal prognosis in patients with first-onset class III/IV lupus nephritis

Asaka Hachiya [1], Munetoshi Karasawa [1], Takahiro Imaizumi [1,2], Noritoshi Kato [1], Takayuki Katsuno [3], Takuji Ishimoto [1], Tomoki Kosugi [1], Naotake Tsuboi [4], Shoichi Maruyama [1*]

[1] *Department of Nephrology, Nagoya University Graduate School of Medicine, Nagoya, Aichi, Japan*
[2] *Center for Advanced Medicine and Clinical Research, Nagoya University Hospital, Nagoya, Aichi, Japan*
[3] *Department of Nephrology and Rheumatology, Aichi Medical University, Nagakute, Aichi, Japan*
[4] *Department of Nephrology, Fujita Health University Graduate School of Medicine, Toyoake, Aichi, Japan*

***Corresponding author:**
E-mail: marus@med.nagoya-u.ac.jp

Supplementary Table S1. Correlations among pathological findings

	Activity index	Scores of components of activity index						Chronicity index	Scores of components of chronicity index			
		Endocapillary hypercellularity	Neutrophils/ karyorrhexis	Hyaline deposits	Fibrinoid necrosis	Cellular/ fibrocellular crescents	Interstitial inflammation		Global/ segmental sclerosis	Fibrous crescents	Interstitial fibrosis	Tubular atrophy
**Activity index**	1.00											
Endocapillary hypercellularity	0.54	1.00										
Neutrophils/karyorrhexis	0.48	0.47	1.00									
Hyaline deposits	0.67	0.37	0.26	1.00								
Fibrinoid necrosis	0.35	0.11	0.20	0.06	1.00							
Cellular/fibrocellular crescents	0.84	0.23	0.47	0.13	0.21	1.00						
Interstitial Inflammation	0.43	-0.13	0.04	-0.03	-0.01	0.37	1.00					
**Chronicity index**	0.28	-0.23	-0.10	-0.07	-0.02	0.24	0.91	1.00				
Global/segmental sclerosis	-0.12	-0.28	-0.28	-0.17	0.01	-0.09	0.37	0.62	1.00			
Fibrous crescents	0.06	-0.07	-0.13	0.03	-0.04	0.03	0.32	0.50	0.41	1.00		
Interstitial fibrosis	0.36	-0.18	-0.03	-0.02	-0.01	0.31	0.93	0.95	0.40	0.29	1.00	
Tubular atrophy	0.37	-0.19	0.01	-0.06	-0.03	0.33	0.95	0.95	0.38	0.31	0.98	1.00

Correlations are described by using Spearman correlation coefficients.

Supplementary Table S2. Adverse events after the initiation of induction therapy

	All (N = 91)	eGFR < 60 (N = 42)	eGFR >= 60 (N = 49)	P value
Cardiovascular disease, N (%)	2 (2)	1 (2)	1 (2)	0.81
Cerebrovascular disease, N (%)	4 (4)	3 (7)	1 (2)	0.17
Femoral head osteonecrosis, N (%)	11 (12)	6 (14)	5 (10)	0.55
Steroids-induced diabetes, N (%)	37 (41)	22 (52)	15 (31)	0.035
Gastric ulcer, N (%)	2 (2)	2 (5)	0 (0)	0.12
First infectious disease requiring hospitalization, N (%)	16 (18)	7 (17)	8 (16)	0.97
Cause of infection		Pneumonia (3), Gastroenteritis (1), Pyelonephritis (1) Cellulitis (1), Sepsis (1)	Pneumonia (2), Gastroenteritis (3), Pyelonephritis (1), CMV infection (1), Cholecystitis (1),	
Herpes zoster or cytomegalovirus infection, N (%)	31 (34)	16 (38)	15 (31)	0.45
Cancer, N (%)	4 (4)	3 (8)	1 (2)	0.24
Cause of cancer		Vulvar cancer (1), Lung cancer (1), Stomach cancer (1)	Malignant lymphoma (1)	

N, number
